# 肺癌中*EML4*-*ALK*融合基因的存在状态及生物学功能

**DOI:** 10.3779/j.issn.1009-3419.2012.02.09

**Published:** 2012-02-20

**Authors:** 红雨 刘

**Affiliations:** 300052 天津，天津医科大学总医院，天津市肺癌研究所，天津市肺癌转移与肿瘤微环境实验室 Tianjin Key Laboratory of Lung Cancer Metastasis and Tumor Microenvironment, Tianjin Lung Cancer Institute, Tianjin Medical University General Hospital, Tianjin 300052, China

肺癌是当前世界上最常见的恶性肿瘤，主要包括小细胞肺癌（small cell lung cancer, SCLC）和非小细胞肺癌（non-small cell lung cancer, NSCLC）两类，而NSCLC约占肺癌总数的80%。尽管外科手术技术不断提高，化疗新药不断上市并进入临床应用，但肺癌患者的预后仍然很差，原因是大多数NSCLC患者确诊时已处于晚期，失去了手术治疗的机会。同时部分临床诊断为早期的肺癌患者，手术后发生复发转移。长期以来，化疗在晚期NSCLC的治疗中占据主要地位，1995年进行的*meta*分析显示，以铂类为基础的化疗相对于支持治疗可以提高患者存活率^[1]^。20世纪90年代，第三代化疗药物有了较大进展，多西紫杉醇、紫杉醇、长春瑞滨、吉西他滨等药物应用于肺癌患者。然而，以铂类为基础的化疗方案在晚期（Ⅲb或Ⅳ）NSCLC患者中，缓解率（response rate, RR）为30%-40%，中位生存期仅为8个月-10个月^[2]^。如何更好而有效地改善NSCLC患者的生存率和生存时间，提高治疗效果，成为临床和科研工作者的一个研究方向和目标。

近年来，随着分子生物学技术的发展，产生了一些针对细胞特定分子的分子靶向治疗药物，这些药物针对性强，能特异性地杀伤肿瘤细胞。其中，针对表皮生长因子受体酪氨酸激酶抑制剂（epidermal growth factor receptor-tyrosine kinases inhibitors, EGFR-TKI）的分子靶向治疗受到了极大的关注。目前，有两种EGFR-TKI（厄洛替尼和吉非替尼）成功在中国上市，其药物特点是口服、高效、高特异性，病人耐受性好，无骨髓抑制和神经毒性，能明显延长敏感患者生存期^[3]^，改善患者生活质量。但是，相关的临床研究^[4, 5]^结果显示吉非替尼对东方人NSCLC的有效率为25%-35%，对西方人的有效率仅为8%-15%，临床研究表明腺癌、女性、非吸烟、亚裔的NSCLC患者获益明显。

2010年，日本学者^[6]^研究了具有EGFR 19外显子缺失突变和21外显子L858点突变的病例，比较单纯使用吉非替尼组与单纯使用顺铂加紫杉醇组的生存时间后发现，在具有突变的患者中，使用吉非替尼者的生存时间较单纯使用化疗药长（9.2个月 *vs* 6.3个月），而在不具有EGFR外显子突变的非吸烟腺癌患者中，单纯使用吉非替尼者的生存时间也较单纯使用铂类化疗药物的时间延长。因此，在亚洲的非吸烟腺癌患者中，使用EGFR-TKI靶向药物吉非替尼的疗效优于传统的化疗，这无疑是肺癌治疗史上的一个巨大的进步。

然而，2007年，日本学者在腺癌患者中发现了一个亚群，这部分患者具有人类棘皮动物微管相关蛋白样4（echinoderm microtubule-associated- protein-like 4, EML4）和人类间变性淋巴瘤激酶（anaplastic lymphoma kinase, ALK）重排形成的融合基因，即*EML4*-*ALK*融合基因，对EGFR-TKI治疗和传统的化疗不敏感，而可能对ALK抑制剂敏感^[7]^。后来的研究发现，具有EML4-ALK重排的患者有其独特的临床病理生理特征，提示其治疗有别于一般的腺癌，本文就*EML4*-*ALK*融合基因的发现及研究现状作一综述。

## 1 *EML4*-*ALK*融合基因的发现

2007年日本学者Soda等^[7]^在一名62岁男性吸烟肺腺癌患者手术标本中扩增出一个由3, 926个碱基组成的cDNA片段，编码一个由1, 059个氨基酸组成的蛋白质，该蛋白的氨基端（残基1-496）是EML4编码蛋白的一部分，而羧基端（残基497-1, 059）则由ALK编码蛋白的一部分构成，提示这个cDNA片段由是EML4和ALK的融合形成。此病例的*EGFR*和*KRAS*基因均为野生型。如图 1所示，该融合基因定位于2号染色体的短臂上（2p21和2p23），其5’端为EML4的片段，3’端为ALK的片段，由倒置后的*EML4*基因片段与残余的ALK片段连接。该融合基因拥有*EML4*基因中的basic区域、疏水的棘皮动物微管相关蛋白区（hydrophobic echinoderm microtubule-associated protein-like protein, HELP）以及部分WD重复区（后两部分在部分亚型中缺失）和*ALK*基因中的Kinase功能区；此外在75个病例中发现了另外5例也具有*EML4*-*ALK*融合基因，因此，提示具有*EML4*-*ALK*融合基因的患者可能为NSCLC的一个特殊群体。

**1:**
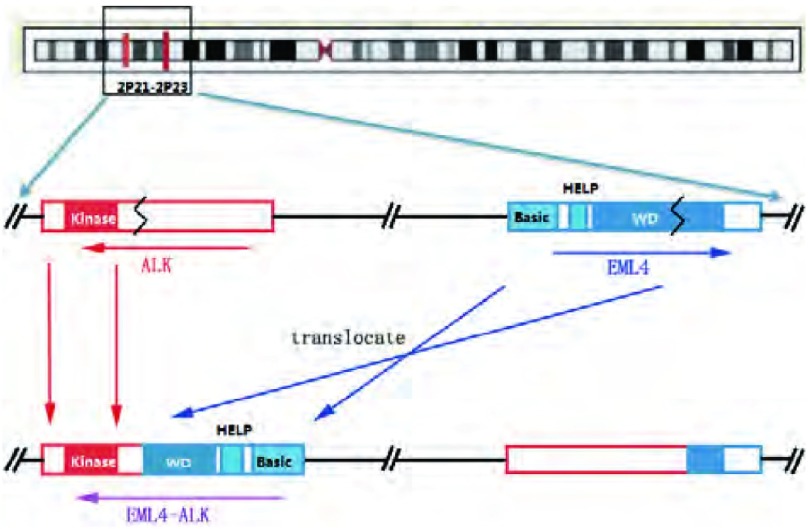
*EML4*-*ALK*融合基因形成示意图 Schematic representation of fusion junctions and flanking sequences of the *EML4*-*ALK* fusion gene. Fusion of the N-terminal portion of EML4 (comprising the basic region, the HELP domain and part of the WD-repeat region) to the intracellular region of ALK (containing the tyrosine kinase domain).

目前，已至少发现了14种EML4-ALK的变异体。这些变异体均有功能，均为EML4的不同外显子与ALK的20外显子相融合。变种1（V1），为EML4的第13外显子与ALK的第20外显子相连；变种2（V2），为EML4的第20外显子与ALK的第20外显子相连^[7]^；变种3（V3），有两个亚变种（V3a/b），V3a为EML4的第6外显子与ALK的第20外显子相连，而V3b为EML4的第6外显子加上一个33 bp的小片段再与ALK的第20外显子相连^[8]^；变种4（V4），为EML4的第14外显子加上一个11 bp的小片段再与前面缺失49 bp的ALK的第20外显子相连；变种5（V5），有两个亚变种（V5a/b），V5a为EML4的第2外显子与ALK的第20外显子相连，而V3b为EML4的第2外显子加上一个117 bp的小片段再与ALK的第20外显子相连^[9]^；变种6（V6），为EML4的第13外显子加上一个49 bp的小片段再与ALK的第20外显子相连；变种7（V7），为EML4的第14外显子与前面缺失12 bp的ALK的第20外显子相连；变种8（“V4”），为EML4的第15外显子缺失了后面19 bp再与前面缺失20 bp的ALK的第20外显子相连；变种9（“V5”），为EML4的第18外显子与ALK的第20外显子相连^[10]^。

## 2 *EML4*-*ALK*融合基因阳性患者的病理生理学特征

几乎所有研究均显示*EML4*-*ALK*融合基因主要存在于NSCLC中，其出现频率为5%-10%。研究显示*EML4*-*ALK*融合基因阳性患者多为不吸烟或少吸烟的腺癌患者，多伴随印戒细胞样改变，与*EGFR*突变相比，*EML4*-*ALK*融合基因多出现于男性，较*EGFR*突变者年轻，在亚洲人群中出现的比例较大；与*EGFR*突变和*KRAS*突变多不会同时出现，即具有*EML4*-*ALK*融合基因的患者多为*EGFR*和*KRAS*野生型，而*EGFR*和/或*KRAS*突变的患者多不具有*EML4*-*ALK*融合基因^[11, 12]^。EGFR-ALK患者对EGFR-TKI治疗不敏感，表现为对TKI治疗的原发性耐药；比较EML4-ALK阳性组与阴性组后发现两组患者对铂类药物的治疗效果无明显差异，总的生存时间也无统计学差异^[13]^。

## 3 *EML4*-*ALK*融合基因可能的生物学功能

*EML4*-*ALK*融合基因的发现者Soda等将携带*EML4*-*ALK*基因的质粒转染小鼠胚胎成纤维细胞3T3，而使后者获得了致瘤性，接种于裸鼠皮下后成瘤。而在携带EML4-ALK的所有转基因小鼠的肺组织中，自发性形成数百个腺癌病灶。这种转基因的小鼠在口服ALK激酶的抑制剂后，肿瘤均趋于消失；而在静脉注射转染EML4-ALK的3T3细胞后，小鼠均由于呼吸衰竭而死亡，而使用ALK激酶抑制剂后，能明显延长这些小鼠的生存时间。使用特异的siRNA沉默*EML4*-*ALK*融合基因后，能抑制相关肿瘤细胞的生长^[14]^。这些均提示*EML4*-*ALK*基因在肺癌的形成过程中起着重要的作用。

研究者研究了*EML4*-*ALK*融合基因的各个功能区，其中，*EML4*基因片段中的basic区及*ALK*基因的kinase区在各个亚型中均包含。后者为*ALK*基因的膜内催化区域，其激活后通过相关信号通路与细胞增殖、存活、迁移密切相关。*EML4*基因片段扮演着能使该融合基因产物蛋白二聚化的功能，因为*ALK*基因的膜内催化区域能在形成二聚体时激活，其异常激活导致细胞的癌变。Soda等^[7]^构建缺失EML4 Basic区、HELP区、WD区质粒的3T3细胞，接种裸鼠后发现缺失EML4 Basic区完全不能成瘤，而缺失HELP区和WD区均能成瘤，但瘤体较小，提示各功能区在肺癌的转化过程中均发挥作用，但Basic区的作用可能最为重要。

对于*EML4*-*ALK*融合基因信号通路的研究，与其它*ALK*基因重排的融合基因一样，都是对*ALK*基因信号通路的研究，都是通过激活*ALK*基因的膜内催化区域达到细胞癌变的效果。目前研究发现其与STAT3、AKT/PI3K及RAS/ERK三条信号通路有关系。转录因子C/EBPβ（CCAAT/enhancer binding protein beta）具有提高细胞增殖和生存的能力。*ALK*基因通过STAT3信号通路提高C/EBPβ的mRNA及蛋白水平，还通过ERK1/2磷酸化C/EBPβ的第235位苏氨酸激活C/EBPβ蛋白^[15]^。研究^[16]^发现ALK也可以通过SHH/GLI1信号通路发挥作用，且可激活AKT/PI3K信号通路稳定GLI1蛋白。

## 4 *EML4*-*ALK*融合基因的检测方法

自从该融合基因发现以来，为寻找更简便和更准确的方法，研究人员做了各方面大量的研究。目前最常用的方法是反转录PCR（RT-PCR）。RT-PCR是一种很灵敏的技术，可以检测很低拷贝数的RNA。RT-PCR广泛应用于遗传病的诊断，并且可以用于定量监测某种RNA的含量。利用手术切除标本或其他标本，提取总RNA后进行RT-PCR，对PCR产物进行电泳以确定是否含有*EML4*-*ALK*融合基因。通过对各种不同亚型进行专门引物的设计，可以分辨出各种不同亚型的*EML4*-*ALK*融合基因。

荧光原位杂交技术（fluorescent in situ hybridization, FISH）也是常用的方法。FISH是利用荧光标记的特异核酸探针与细胞内相应的靶DNA分子或RNA分子杂交，通过在荧光显微镜或共聚焦激光扫描仪下观察荧光信号，来确定与特异探针杂交后被染色的细胞或细胞器的形态和分布，或者是结合了荧光探针的DNA区域或RNA分子在染色体或其它细胞器中的定位。通过对*EML4*基因及*ALK*基因进行荧光标记，在荧光显微镜下查看这两种基因的位置关系判断是否有染色体易位，以确定是否有EML4-ALK融合基因^[17]^。

Western blot的方法也是检测*EML4*-*ALK*融合基因的方法之一。Western blot采用的是聚丙烯酰胺凝胶电泳，经过PAGE分离的蛋白质样品，转移到固相载体（例如硝酸纤维素膜NC膜）上，固相载体以非共价键形式吸附蛋白质，且能保持电泳分离的多肽类型及其生物学活性不变。以固相载体上的蛋白质或多肽作为抗原，与对应的抗体起免疫反应，再与酶或同位素标记的第二抗体起反应，经过底物显色或放射自显影以检测电泳分离的特异性目的基因表达的蛋白成分。Western blot主要用来确定*EML4*-*ALK*融合基因是否表达及表达情况。

除了以上几种常用的方法外，还有其它各种常规或新的方法。Zhang等^[18]^使用了一种RACE-coupled PCR的方法，也很好地检测出了*EML4*-*ALK*融合基因阳性的样本。Takeuchi等^[19]^发现了一种新的检测*ALK*相关融合基因的免疫组化方法-iAEP（antibody-enhanced polymer）。该法将ALK蛋白的不同区域分别做出相应的特异性抗体，然后用这些抗体特异检测标本中ALK蛋白不同区域的表达情况。若5’端的区域与3’端的区域表达差异较大，则说明有ALK融合基因。表 1列出了文献^[7, 9, 11, 13, 14, 18-25]^报道的检测EML4-ALK的方法及阳性率。

**1:** 文献所用检测EML4-ALK的方法及阳性率 EML4-ALK fusion gene previously reported by the literatures in lung cancer

Author	EML4-ALK（%, *n*/*N*）	Area	Method
Soda^[7]^	6.67%（5/75）	Japan	RT-PCR
Takeuchi^[9]^	4.35%（11/253）	Japan	RT-PCR
Jokoji^[11]^	3.15%（8/254）	Japan	FISH
Shaw^[13]^	13.48%（19/141）	World	FISH
Lin^[14]^	11.32%（12/106）	US	FISH，exon array，RT-PCR
Zhang^[18]^	11.65%（12/103）	China	RACE-coupled PCR
Takeuchi^[19]^	6.15%（8/130）	Japan	iAEP，RT-PCR
Fukuyoshi^[20]^	0.96%（1/104）	Japan	RT-PCR
Koivunen^[21]^	2.62%（8/305）	US (138), Korea (167)	RT-PCR
Inamura^[22]^	3.03%（11/363）	Japan	RT-PCR
Martelli^[23]^	7.50%（9/120）	Italy, Spain	RT-PCR
Takahashi^[24]^	1.60%（5/313）	Japan	RT-PCR
Wong^[25]^	4.89%（13/266）	China	RT-PCR

## 5 展望

目前在NSCLC中*EML4*-*ALK*融合基因的亚型已经有14种。Lin等^[14]^发现一种EML4的21外显子与ALK的20外显子融合而成的新亚型，但仅在大肠癌中出现，并未在肺癌中检测到，或许更敏感的检测方法或更大样本量的检测能发现更多新的亚型。

现在对*EML4*-*ALK*融合基因在信号转导通路方面的研究还刚开始，相信这部分的研究将是以后的重点。如果信号通路明确的话，可以设计相应的药物，这将对治疗有极大的帮助。实验中通过对*EML4*-*ALK*融合基因阳性的细胞系及*EML4*-*ALK*融合基因阳性的小鼠进行一些尝试性的治疗，观察细胞系的生长情况及小鼠肿瘤的生长情况，发现其对吉非替尼耐药，对铂类药物低反应，但ALK抑制剂对其有效，能明显抑制肿瘤的生长^[13, 26]^。

由于目前在临床上相关特征并无超大样本的统计资料，对一些特征还是比较难以把握，并且一些更常规高效的检测方法有待研究，*EML4*-*ALK*融合基因检测真正应用于临床还有一定的距离。

综上所述，*EML4*-*ALK*融合基因存在于NSCLC中，很有可能成为在*EGFR*突变与*KRAS*突变之外的另一个重要的分子靶点，进一步研究可能会展现出与NSCLC的发生、发展、治疗及评估等方面相关的新领域。
